# Health and physical fitness profiling of working population: Sport4Health 2021

**DOI:** 10.1186/s12919-021-00216-5

**Published:** 2021-05-17

**Authors:** Nikola Todorovic, Valdemar Stajer, Bojana Harrison, Darinka Korovljev, Nebojsa Maksimovic, John van Heel, Damjan Pintar, Hasan Ibric, Milko Kralski, Igor Jukic, Sophie Kekic, Sergej M. Ostojic

**Affiliations:** Faculty of Sport and Physical Education, University of Novi Sad, Lovcenska 16, Novi Sad, 21000 Serbia

**Keywords:** SPORT4H, Physical activity, Health, Fitness, Workplace

## Abstract

Sport4Health Network (SPORT4H) is a multidisciplinary project co-funded by the European Union Erasmus+ programme aimed to encourage participation in physical activity in working population. SPORT4H includes educational and instructional activities that provide top-notch knowledge on various physical activities that may have an additional benefit to improve healthy lifestyle behaviours across workforce. The aims of Sport4Health 2021 e-symposium organized from 22nd to 23th March 2021 were to: (1) summarize data collected during this project through evaluation of health and fitness profiles for over 40,000 employees from all Sport4HealthNet countries (Belgium, Bulgaria, Croatia, Netherlands, Serbia and Slovenia); (2) discuss the applicability of user-friendly guidelines for physical activity at workplace and e-learning module that includes multicomponent interventions with innovative activities; (3) share experiences from different partners about the effects of educational interventions in specific working environment; and, (4) overview challenges identified during the implementation of interventions at work settings. The Sport4Health 2021 e-symposium facilitated networking between partner institutions, provided practical information for extensive public education that advances physical activity at workplace, and capacitated interaction and recruitment of end-users through e-learning modules and guidelines.

## Background

The World Health Organization (WHO) European Policy Framework and Strategy for the twenty-first century (Health 2020) prioritizes investing in health promotion programmes by utilising existing social networks such as the one in the workplace [1]. As workers generally spend more time in the workplace than any other location, the workplace can have a direct impact on workers’ physical, mental, economic and social health. The European Network for Workplace Health Promotion (ENWHP) has defined workplace health promotion in their Luxembourg Declaration as the combined efforts of employers, employees and society to improve the health and wellbeing of people at work [2]. The European Agency for Safety and Health at Work (EASHW) find that well-implemented workplace health promotion can lead to improved working environment and a decrease in absenteeism, and therefore recommend that policies continue to emphasise the importance of workplace health promotion [3]. The most recent Eurobarometer on Sport and Physical Activity identifies that 13% of physical activities take place at work, with no changes seen as compared to previous report in 2013 [4].

## Sport4Health network project

Sport4Health Network (SPORT4H) is a transnational multi-year educational and research project that is co-funded by the European Union Erasmus+ programme. SPORT4H joins together health and exercise professionals from six European countries (Belgium, Bulgaria, Croatia, Netherlands, Serbia, and Slovenia) in an aim to encourage participation in sport and physical activity among employees all around Europe. Among other objectives, the project strives to create better access and more opportunities in people’s everyday lives to engage in exercise and maintain a healthy lifestyle [5]. In an aim to summarize data collected during this project through evaluation of health profiles for over 6000 of employees from all Sport4HealthNet countries, discuss the applicability of user-friendly guidelines for physical activity at workplace and e-learning module that includes multicomponent interventions with innovative activities, share experiences from different partners about the effects of educational interventions in specific working environment and overview challenges identified during the implementation of interventions at work settings, we organized a two-day e-symposium from 22nd to 23th March 2021, and brought together all partners, public health and exercise experts, and policy administrators. Other details about the project are available at SPORT4H webpage (https://sport4healthnet.eu/).

## Summary of presentations

### A1: advancing health-enhancing physical activity at workplace: an overview

Physical activity (PA) at workplace can positively impact various wellbeing outcomes yet developing and implementing exercise programs that are straightforward, time-efficient and widely applicable remains a notable public health challenge. We recently critically evaluated evidence on stretching and resistance exercise programs targeted at working population in an aim to identify knowledge gaps and future areas of research and application [5]. Only a handful of studies evaluated the applicability of non-traditional physical activity programs in working population. However, we identified a moderate-to-strong link between non-traditional physical activity programs (such as flexibility and strengthening exercise) at the workplace and several indices of health-related physical fitness. Resistance exercise turned out to be superior to other exercise interventions analysed, with low-volume high-repetition exercise positively affecting work performance, musculoskeletal disorders, and health-related quality of life in employees. The recommendation includes exercise at least 3 times per week for over 8 weeks. Besides, screening protocols should employ health-related questionnaires, adopting a progressive training load, and prescribing training programs to individual participant’s needs. Implementing non-traditional PA programs aimed to improve health-related physical fitness and counteract sedentary behaviour at workplace might therefore be of utmost importance to contribute to health promotion in this sensitive population [5].

### A2: SPORT4H health-related physical fitness evaluation and prescription manual

Having enough physical activity to advance health and wellbeing remains a challenging task for Europeans of all ages and backgrounds [4]. Many general guidelines and educational materials that promote healthy exercise are already available, yet somehow a majority of Europeans do not meet the physical activity recommendations while staying sedentary and physically inactive. Among others, members of the workforce tend to spend more time sitting while having fewer opportunities (and time) to exercise. Therefore, this sensitive population requires an attentive approach of the academic community to address specific needs for physical activity and provide easy-to-digest instructions to promote healthy behaviours both at the workplace and at home. The manual *Stretching and Strengthening at Work* that our consortium developed during the SPORT4H project has been designed as a possible step forward, considering it is a guidebook that helps test the workforce lifestyles but also provides an illustrative document that contains many exercises for improving flexibility and muscular strength at workplace [6]. As one of the main intellectual outputs and deliverables of SPORT4H Network, the manual is intended to assist employees in improving health-related physical fitness by taking part in simple, short-term, specific and convenient exercises either at the workplace, at home or during leisure. Exercises described in the manual are primarily designed for healthy adults; one should check with a health care provider before beginning exercise regimen to make sure he/she is medically able to participate. Besides the detailed description of how to get and stay active for health, and how to test health-related physical fitness at work, the manual contains 76 exercises and alternatives directed to improve flexibility and muscular strength during work hours. Finally, *Stretching and Strengthening at Work* also includes recommendations for special populations, and many exercise tips and advices to promote being active both at workplace, at home and during leisure.

### A3: development of training courses for health and fitness professionals

Training courses developed under SPORT4H project were designed to motivate and support healthy lifestyles among company employees as well as to further educate well qualified health and fitness professionals by improving their knowledge and acquiring certain skills in this field through a direct work with employees. The goal of this initiative was to provide necessary training to health and fitness professionals according to the Sport4H methodology described in the manual *Stretching and Strengthening at Work* [6], in order to integrate the knowledge through various processes of creating in-service exercise programs, as well as various strategic activities, which result in favourable health outcomes, workplace quality, well-being and better productivity of employees. Upon a successful completion of the courses, health and fitness professionals were trained to effectively deliver a certain standard of knowledge in this field, and design and implement programs for improving health-related physical fitness and counteract sedentary behaviour at workplace in various private and public companies and organizations. The attendees learned how to work with employees on site, how to use the manual and implement everyday, short (~ 5 min) stretching and strengthening exercises, assess and evaluate health profiles of the employees and promote healthy lifestyles among working population.

### A4: recruitment of participants: data analyses and challenges

Participants recruitment for intervention/research is often the most challenging and resource-intensive aspect of a study. First of all, participants must voluntarily agree to take part in the project/intervention. Ethical principles must be adhered to and potential participants can decline to take part*.* All participants in our project gave an informed consent, in which a fully informed individual voluntarily decides whether or not to take part as an intervention/research participant. Informed consent includes information such as the nature of the intervention/research, benefits, and potential risks. It is well known that 2020 was a pandemic year due to the spread of the coronavirus. Thus, we had to face the quarantine, restricted working time, and in general, employees were more conservative to take part in any kind of intervention. A strategic plan was made, and with good management, the educational interventions with the employees were conducted, convincing them that the inclusion in physical activity and the benefits it brings outweigh the potential risks*.* Participants’ recruitment was conducted in all six partner European countries (Belgium, Bulgaria, Croatia, Netherlands, Serbia and Slovenia). The project’s goal was to recruit as many employees as possible, regardless of their position within a company. Over 40,500 employees, from teachers, directors and engineers, to IT employees, mechanics and locksmiths, were recruited at the beginning of the project. A distribution of study participants at baseline has been depicted in Table 1. Of those, 3847 participants were evaluated for at least one outcome at the follow up, with complete data for SPORT4H health-related physical fitness profiles were available and analyzed for 1671 employees. Data were analyzed using SPSS Statistics for Mac Version 24.0 (IBM, Armonk, NY),

**Table 1:** Distribution of study participants across partner countries

	No. of participants
Serbia	26,392
Croatia	7762
Netherlands	2000
Slovenia	1996
Bulgaria	1350
Belgium	1000
TOTAL	40,500

### A5: SPORT4H testing kit: from physical fitness to the health-related quality of life

The definition of health-related physical fitness involves exercise activities that a person can do in order to try to improve her or his physical health and stay healthy. The evaluation of health-related physical fitness in different populations, including workforce, is a fairly common practice by a sport-for-health professional. There are several reasons to evaluate each component of health- related physical fitness. Some of the reasons may include education of employees about their current level of health-related physical fitness, utilizing data from the evaluation to personalize physical activity programs, provide baseline and follow-up data to evaluate exercise programs at workplace, and motivate employees towards more specific action. For SPORT4H project, we developed a complex set of testing procedures that include the assessment of several elements of health-related physical fitness, such as body composition (e.g., body mass index, waist circumference, and fat mass), cardiorespiratory endurance (including maximal oxygen uptake), muscular fitness (e.g.*,* handgrip strength and abdominal muscle endurance), and flexibility (sit-and-reach test). Advanced assessment also incorporates the level of physical activity at home and at work (using International Physical Activity Questionnaire), health-related quality of life (Short Form 12 Health Survey, SF-12), and health-promoting lifestyle habits of workforce (Health-Promoting Lifestyle Profile, HPLP-II). This set of field tests is simple, convenient and easy-to-conduct in various workplace settings, making it highly applicable for many purposes.

### A6: health-related physical fitness profiles in SPORT4H employees

A subsample of 1671 workers (1369 men and 302 women, age 41.2 ± 11.2 and 39.3 ± 8.2 years, respectively) provided information for all components of health-related physical fitness. The participants belonged to various professions, from manual workers, to office workers, to management positions. The summary of health-related physical fitness profiles is presented in Table 2.

**Table 2:** Health-related physical fitness profiles of study participants. Values are mean ± SD

	Men	Women
Age (years)	41.2 ± 11.2	39.3 _0 8.2
Body mass index (kg/m^2^)	27.6 ± 4.2	24.8 ± 5.1
Waist-to-hip ratio	0.94 ± 0.32	0.76 ± 0.07
Body fat (%)	24.4 ± 6.4	31.4 ± 8.1
Handgrip strength (kg)	97.7 ± 20.5	58.1 ± 13.2
Abdominal endurance (score)	14 ± 10	8 ± 9
Sit-and-Reach test (cm)	22.4 ± 10.7	29.5 ± 9.6
Cardiorespiratory endurance (ml/kg/min)	42.6 ± 7.7	33.1 ± 6.5

**Fig. 1:**
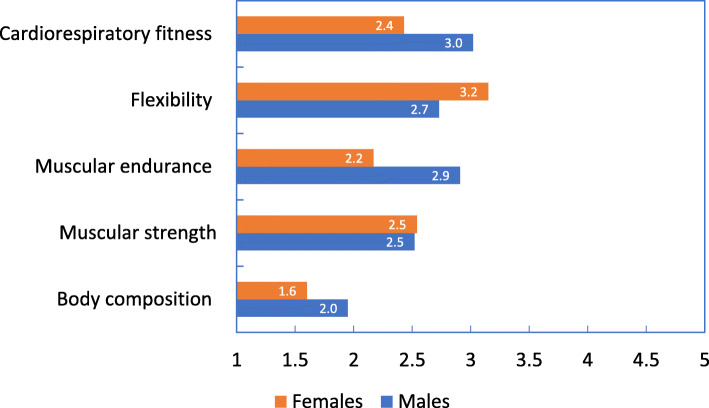
Health-related physical fitness components. Grade 1 represents very poor physical fitness, and grade 5 represents an excellent fitness profile

Overall, workers’ health-related physical fitness was below average comparing to the normative values [7]. Inadequate level of fitness was demonstrated for each component of health-related physical fitness (Fig. 1), with all components below grade 3 (average), except for flexibility in women. The findings call for rather extensive exercise program that should tackle health-related physical fitness of employees in an aim to improve general health and wellbeing.

### A7: educational intervention and practical demonstrations: building the case

The education of the employees in companies was certainly one of the most important project activities and one of the basic goals of the project. This process consisted of theoretical and practical work in terms of theoretical education and practical intervention on the basic principles of a healthy lifestyle and physical activity at the workplace. The implementation of the employee training took place simultaneously in all six partner countries participating in the project, according to the SPORT4H modus operandi. A total of 18 previously trained and well-qualified health and fitness professionals conducted the practical instruction and on-site training by going to different types of companies and by collaborating with employees at their workplace. The educational intervention (~ 45 min per session per company) consisted of training the employees to apply the stretching and strengthening exercise properly. After the employees received the basic information, they had a practical demonstration followed by the three-month intervention. The employees were required to exercise at the workplace for 15 min per session, three times a week, following both the e-format and printed *Stretching and Strengthening at Work* guidelines. After the 3-month intervention, the participants were re-evaluated for health and fitness profiles, and all employees were informed about the changes during the three-month period, as well as how to keep on being active at the workplace.

### A8: effects of SPORT4H intervention on health-related physical fitness profiles

**Fig. 2:**
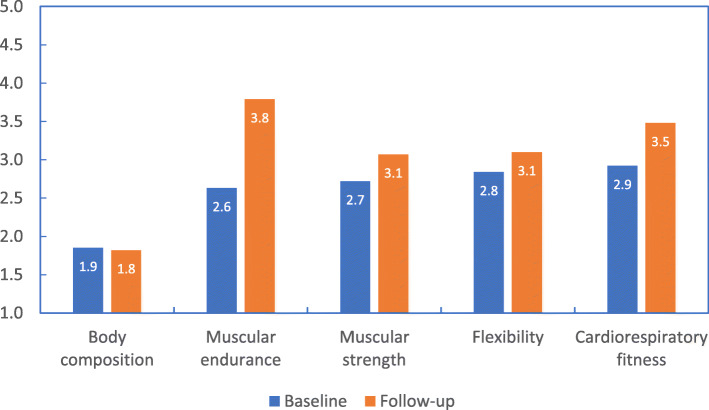
Changes in health-related physical fitness during the study. Grade 1 represents very poor physical fitness, and grade 5 represents an excellent fitness profile

The main aim of this component of SPORT4H project was to evaluate the changes in muscular fitness, cardiorespiratory fitness and body composition after the on-site intervention in male and female workers (see Presentation A3). A 3-month strength and stretching exercise program (e.g., 3 times per week, 15–20 min per session) has been conducted with 3847 workers, with both baseline and follow-up health-related physical fitness profiles available for 1671 participants. We found that the program induced significant changes in most health-related physical fitness components in SPORT4H participants, with changes from baseline to 3-month follow up depicted in Fig. 2.

Physical fitness profiles among employees improved in four main fitness components (e.g.*,* muscle strength and endurance, cardiorespiratory fitness, flexibility), while body composition remained similar to the pre-intervention level. Therefore, an increased awareness of physical activity benefits, using workplace exercise interventions and health promotion campaigns such as SPORT4H, can contribute to a beneficial change in health-related physical fitness profiles.

### A9: effects of SPORT4H intervention on lifestyle behaviours

Health promotion is a healthcare strategy that implies changes in behaviour and the adoption of patterns that support good health in order to enhance the quality of life. Besides tackling with health-related physical fitness, SPORT4H also strives to improve people’s awareness of the importance of life habits both at workplace and at home, and during leisure. Here, we presented the effects of SPORT4H 3-month educational intervention on health-promoting lifestyle profile (HPLP-II), a multi-component tool that assesses 6 dimensions of lifestyle, including physical activity, health responsibility, nutrition, spiritual growth, interpersonal relationships and stress management in our sample of SPORT4H employees from six partner countries (Table 3).

**Table 3:** Changes in health-promoting lifestyle profile during the study

	Baseline	Follow up	*P*
Stress management (score) *	2.5 ± 0.5	2.3 ± 0.5	< 0.01
Physical activity (score)	2.0 ± 0.5	2.3 ± 0.6	< 0.01
Health responsibility (score)	2.1 ± 0.5	2.5 ± 0.4	< 0.01
Nutrition (score)	2.4 ± 0.4	2.8 ± 0.5	0.34
Spiritual growth (score)	2.8 ± 0.5	2.9 ± 0.4	< 0.01
Interpersonal relationships (score) *	3.0 ± 0.4	2.5 ± 0.4	< 0.01
Total score (score) *	2.5 ± 0.3	2.6 ± 0.3	< 0.01

Asterisk (*) indicates components where lower scores mean better lifestyle profiles

The results show significant impact of the intervention for almost every measured aspect of life habits, except for spiritual growth component of the HPLP-II questionnaire. These findings highlight the importance of SPORT4H intervention among employees. Although SPORT4H intervention affected most of the measured variables, future studies should perhaps include individualized exercise programs and scrutinize its effectivity in this sensitive population.

### A10: effects of SPORT4H intervention on health-related quality of life

Health-related quality of life (HRQL) is a broad multidimensional concept that usually includes subjective evaluations of both positive and negative aspects of life. It contains a variety of components such as physical, mental, emotional, and social functioning. To determine the effects of a 3-month intervention, we conducted a SF-12 survey in SPORT4H cohort (see presentation A6), the results are depicted in Table 4.

**Table 4:** Changes in SF-12 health-related quality of life during the study

	Baseline	Follow up	*P*
General health	56 ± 28	60 ± 26	0.03
Limited moderate activities	71 ± 34	70 ± 35	0.96
Climbing several flights of stairs	69 ± 39	71 ± 35	0.26
Accomplished less than would like: physical health	69 ± 46	78 ± 42	0.00
Limited in work or other activities	69 ± 46	76 ± 43	0.01
Accomplished less than would like: emotional health	79 ± 42	78 ± 42	0.57
Didn’t do work as carefully as usual	74 ± 44	79 ± 41	0.06
Pain interfering with normal work	72 ± 28	72 ± 27	0.51
Felt calm and peaceful	64 ± 22	61 ± 20	0.02
Have a lot of energy	63 ± 22	60 ± 21	0.00
Felt downhearted and blue	67 ± 19	68 ± 21	0.58
Health problems interfered with social activities	68 ± 26	71 ± 71	0.18

**Fig. 3:**
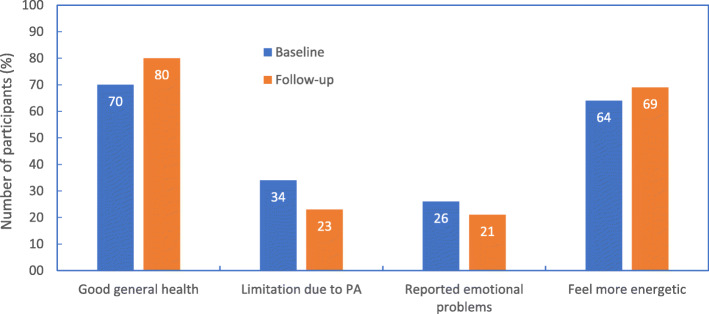
Changes in subdomains of health-related quality of life. PA – Physical activity

SPORT4H intervention improved health-related quality of life in multiple domains. The most significant impact is noticed in everyday activities limited due to the lack of general strength (SF-4). A notable difference was also notified in general health (SF-1) and emotional stability (SF-9 and SF-10). Comparing these results with the worldwide population’s standard mean values, participants in this study were above the 50 percentiles of mean averages for general population [8]. In addition, SPORT4H intervention improved cumulative HRQL subdomains (Fig. 3).

### A11: developing e-learning module: steps in the process

The e-learning module was developed based on the Sport4Health methodology and guidelines that describe different stretching and strength exercises that are appropriate for a typical workplace, with detailed description of exercise execution with regard to different degrees of intensity, duration and number of repetitions, in order to make it available to a wider audience using the online tools. The process of developing of the e-learning videos for the Sport4Health project started with the analysis of the exercises from the guidelines with regards to effectiveness and applicability. Based on this analysis, the exercises at the workplace were grouped into two basic sections: (a) improving flexibility during working hours, and (b) improving muscular strength during working hours. Filming was done with both male and female actors to create more diversity and motivate both genders. Altogether, 68 videos were filmed and edited with written description in English language. The first section contains: 1. Neck and spine exercises (8 videos), 2. Upper extremity stretches (10 videos) and 3. Lower extremity stretches (8 videos). The second section contains: 1. Standing upper body exercises (4 videos), 2. Neck and spine exercises (4 videos), 3. Lower body exercises (9 videos), 4. Mat exercises for glutes and back (4 videos), 5. Mat exercises for abdominals (11 videos), 6. Mat and office exercises for upper body (3 videos), 7. Whole-body exercise at workplace (4 videos). Further three videos were created for special populations. Further to the exercises at the workplace, the e-learning module contains videos showing how to be active at home and during leisure, videos containing recommendations on how to exercise safely, with tips and advices. Special attention was given to creating videos for physical activities for special populations.

### A12: building on-line testing tools for lifestyle behaviours: a case study

An online research was conducted within the Sport4Health project with the basic goal of determining health profiles and lifestyle behaviors of the working population. For the purposes of this online survey, two standardized questionnaires were identified and adapted to an e-format, International Physical Activity Questionnaire [9], and Health-Promoting Lifestyle Profile [10]. The online e-format tests were made using 1KA software, so they would be available in electronic form and translated into a total of 8 languages, which are officially used in the countries participating in the project consortium: Slovenian, Dutch, German, Bulgarian, Croatian, Serbian, English and French. 1KA is an open-source application that enables services for online surveys and the development takes place at the Centre for Social Informatics, at the Faculty of Social Sciences, University of Ljubljana. The University of Ljubljana is also the formal owner or addressee of the corresponding intellectual property. After the technical preparation and translation, tests were posted on the web platform www.sport4healthnet.eu for ease of access, in order to conduct the survey and data collection within the working population in all six countries, members of the project team: Belgium, Bulgaria, Croatia, Netherlands, Serbia and Slovenia. The basic idea was to collect data by sending a link with tests to > 1000 e-mail addresses through the network of each project partner country, collecting individual responses at the baseline and the follow-up. Educational intervention was conducted using SPORT4H materials. Based on the analysis of the data obtained from SPORT4H respondents (see presentations A9 and A10), it can be concluded that there have been positive changes related to the adoption of healthy behaviors and habits during working hours. The application of short interventions through Sport4H exercises and education for lifestyle behaviors during working hours can thus be recommended as an effective and safe method for improving the health status of the working population. Online testing tools proved to be quite a useful method for data collection as direct access to companies was rather limited due to the COVID-19 pandemic that affected the implementation of the project activities on site in all six project partner countries.

### A13: SPORT4H implementation using online and offline tools

In order to reach the widest audiences possible, the SPORT4H project results were disseminated through a variety of online and offline tools. The entire process of the development of the instruction manual *Stretching and Strengthening at the Workplace* and its application, together with the study and its results, were presented on the project’s website, as well as through social media, including Facebook, Twitter, Instagram, LinkedIn, and YouTube. Short descriptive video clips were filmed, showing each SPORT4H exercise in detail (https://sport4healthnet.eu/). The Sport4HealthNet mobile app was developed, to enable end-users with an easy approach to the tools necessary to maintain healthy lifestyle. Although the circumstances with the pandemic of the COVID19 were not conducive for a widespread demonstration of the project results in person, a few very successful sports events and scientific forums were still organized, with respect of all the precautionary measures.

## Conclusions

SPORT4H project demonstrates beneficial effects of short-term exercise and educational intervention on health-related physical fitness, lifestyle behaviours, and quality of life in employees across Europe. Educating workforce using a plethora of written, verbal and video media and materials appears to be a rather effective, applicable and suitable tool in driving positive changes in this sensitive population. SPORT4H know-hows perhaps remain as a useful expertise for others to use and built into all lifestyle interventions targeted to working population and beyond. Multidisciplinary and trans-national collaboration established through SPORT4H network enables fruitful exchange of expertise between partners from academia, industry, non-governmental and public entities, and successful overcoming of challenges identified during the implementation of this project.

## Data Availability

Not applicable.

## Abbreviations

ENWHP: European Network for Workplace Health Promotion
EASHW: European Agency for Safety and Health at Work
HPLP-II: Health-promoting lifestyle profile
HRQL: Health-related quality of life
PA: Physical activity
SPORT4H: Sport for Health Network
WHO: World Health Organization
